# Artificial Intelligence Use Among Health Care Professionals in the United States and United Kingdom: Cross-National Survey

**DOI:** 10.2196/100681

**Published:** 2026-10-19

**Authors:** Emre Sezgin, Jennifer Lee, Tomasz Jadczyk, Alysha J Taxter

**Affiliations:** 1 College of Medicine, The Ohio State University, Columbus, OH, 43205, United States; 2 Abigail Wexner Research Institute, Nationwide Children's Hospital, 700 Children's Drive, Columbus, OH, 43205, United States, 1 6146957499; 3 Division of Clinical Informatics, Nationwide Children's Hospital, Columbus, OH, United States; 4 3rd Department of Cardiology, Medical University of Silesia, Katowice, Silesia, Poland; 5 Heart Rhythm Centre, Royal Brompton Hospital, Guy's and St Thomas' NHS Foundation Trust, London, United Kingdom

**Keywords:** artificial intelligence, AI, adoption, cross-national survey, clinician, large language models

## Abstract

This cross-sectional study examined AI use among 524 health care professionals in the United States and United Kingdom, focusing on current applications, access, barriers, and factors that could support adoption. AI use was common but varied across professional and national contexts, with findings highlighting the importance of evidence, organizational guidance, training, privacy safeguards, and integration into clinical workflows for responsible adoption.

## Introduction

Generative AI is increasingly entering clinical work, but evidence remains limited on how health care professionals (HCPs) use AI in routine practice and whether use occurs within sanctioned systems or outside institutional governance [1]. Uptake may differ by demographic and professional factors, raising concerns about equitable integration [2]. To compare adoption patterns across health systems, we surveyed HCPs in the United Kingdom and United States, two high-maturity AI-in-health settings with differing organizational structures [3]. The aim of this paper is to investigate the prevalence, breadth, tasks, access routes, and barriers to workplace AI use, and whether these differ by country and professional role.

## Methods

### Procedure and Analyses

We conducted an anonymous, cross-sectional online survey of HCPs in the United Kingdom and United States, recruited through Prolific (online recruitment platform) between January and March 2026. Eligible respondents were adults currently working in a health care profession. The primary exposure was country, with secondary comparisons by professional role. Breadth of AI use was the count of work-related AI application types endorsed across 9 categories (range 0‐9). Categorical comparisons used chi-square or Fisher exact tests, and univariate linear regression examined associations with breadth, reported as B coefficients with 95% CIs. Analyses used available cases (N=524; denominators vary slightly by item) and were performed in Stata (version 18.0; StataCorp). We followed the STROBE (Strengthening the Reporting of Observational Studies in Epidemiology) reporting guidelines (Table S1 in Multimedia Appendix 1).

### Ethical Considerations

The study was reviewed by the Nationwide Children’s Hospital Institutional Review Board and determined to be exempt (STUDY00005734). Formal informed consent was not required, and participants indicated agreement by voluntarily proceeding with the study. Only deidentified data were collected. Participants were compensated through Prolific ($12/hour).

## Results

Among 524 respondents, 268 (51.1%) were from the United Kingdom and 256 (48.9%) from the United States; 281 (53.6%) were nurses, 111 (21.2%) physicians, and 132 (25.2%) other HCPs, including pharmacists, psychologists, and social workers. Most were female (n=386, 73.7%) and employed full-time (n=390, 74.4%); country groups differed in education (*P*<.001) and experience (*P*=.02) but not age or specialty (Table S2 in Multimedia Appendix 1).

Overall, 397 (75.8%) respondents reported at least 1 workplace AI use in the prior 30 days. The most common uses were documentation support (191/524, 36.5%), literature search/summarization (158/524, 30.2%), and clinical decision support (128/524, 24.4%; Table 1). Nonuse did not differ by country (UK respondents: 65/268, 24.3%; US respondents: 63/256, 24.2%; *P*=.99), and breadth of AI use was similar (B=0.19, 95% CI –0.04 to 0.42; *P*=.11). However, US respondents more often reported clinical decision support use (74/256, 28.9% vs 79/268, 20.1%; *P*=.02), imaging/diagnostics support (27/256, 10.5% vs 16/268, 6.0%; *P*=.05), and coding/billing/prior authorization use (23/256, 9.0% vs 9/268, 3.4%; *P*=.007), whereas UK respondents more often reported translation/language support (34/268, 12.7% vs 12/256, 4.7%; *P*=.001). Documentation, data analysis/quality improvement, translation/language support, and billing/coding/prior authorization uses did not differ by roles.

**Table 1.** AI tool use by respondents (N=524) in the past 30 days by country of residence and professional role. Results are not mutually exclusive (respondents could select multiple tool types).

AI tool type used in the past 30 days	All respondents (N=524), n (%)	Country of residence, n (%)		Professional role, n (%)			*P* value (professional role comparison)
		United Kingdom (n=268)	United States (n=256)	Physician (n=111)	Nurse (n=281)	Other^a^ (n=132)	
Documentation	191 (36.5)	90 (33.6)	101 (39.5)	50 (45.0)	98 (34.9)	43 (32.6)	.10
Clinical decision support	128 (24.4)	54 (20.1)	74 (28.9)	49 (44.1)	62 (22.1)	17 (12.9)	<.001
Literature search/summarization	158 (30.2)	79 (29.5)	79 (30.9)	47 (42.3)	65 (23.1)	46 (34.8)	<.001
Data analysis/quality improvement	84 (16.0)	39 (14.6)	45 (17.6)	17 (15.3)	40 (14.2)	27 (20.5)	.27
Patient messaging drafts	69 (13.2)	34 (12.7)	35 (13.7)	19 (17.1)	32 (11.4)	18 (13.6)	.31
Imaging/diagnostics support	43 (8.2)	16 (6.0)	27 (10.5)	16 (14.4)	24 (8.5)	2 (1.5)	.003
Translation/language support	46 (8.8)	34 (12.7)	12 (4.7)	9 (8.1)	25 (8.9)	12 (9.1)	.96
Coding/billing/prior authorization	32 (6.1)	9 (3.4)	23 (9.0)	10 (9.0)	13 (4.6)	9 (6.8)	.24
Other AI use/not listed	41 (7.8)	25 (9.3)	16 (6.3)	5 (4.5)	27 (9.6)	9 (6.8)	.21
Did not use any AI tools	127 (24.2)	65 (24.3)	62 (24.2)	17 (15.3)	81 (28.8)	29 (22.0)	.02

a “Other” role includes pharmacists, psychologists, and social workers.

Role differences were larger than country differences. Physicians (49/111, 44.1%) more often reported clinical decision support use than nurses (62/281, 22.1%) and other HCPs (17/132, 12.9%; *P*<.001), as well as greater use of literature search (*P*<.001) and imaging/diagnostics tools (*P*=.003). Physicians (17/111, 15.3%) were also less likely to report no AI use than nurses (81/281, 28.8%) and other HCPs (29/132, 22.0%; *P*=.02). In unadjusted univariate models, nurses (B=−0.63, 95% CI −0.92 to −0.33; *P*<.001) and other HCP roles (B=−0.61, 95% CI −0.94 to −0.26; *P=*.001) were associated with lower AI breadth compared with physicians. Graduate education (B=0.85, 95% CI 0.29-1.40*; P*=.003), doctoral education (B=1.03, 95% CI 0.43-1.64; *P*=.004), male sex (B=0.28, 95% CI 0.01-0.55; *P*=.04), and full-time employment (B=0.37, 95% CI 0.10-0.64; *P*=.01) were associated with greater breadth. Black respondents had significantly higher breadth scores compared with Asian respondents (B=0.69, 95% CI 0.17-1.21; *P*=.01). Emergency medicine specialty was associated with marginally greater breadth compared with primary care (B=0.49, 95% CI 0.01-0.98; *P*=.05; Table S3 in Multimedia Appendix 1).

No single barrier predominated for 523 respondents (Table 2; 1 non-respondent removed). The most common barriers were accuracy or reliability (n=78, 14.9%), lack of workplace access (n=77, 14.7%), privacy or security (n=65, 12.4%), and lack of training (n=63, 12.0%); barrier distributions did not differ by country (*P*=.06). Personal, non−employer-provided tools were the most common access mode (n=210, 40.1%), exceeding Electronic health record (EHR)–integrated access (n=128, 24.4%) and employer-approved standalone tools (n=134, 25.6%). Physicians (67/111, 60.4%) were more likely than nurses (87/281, 31.0%) to report personal tool use (*P*<.001), whereas EHR-integrated and employer-approved access did not differ by role. The most endorsed facilitators across 523 respondents were stronger evidence (n=227, 43.3%), organizational policy (n=156, 29.8%), training (n=155, 29.6%), privacy protections (n=149, 28.4%), and EHR integration (n=130, 24.8%).

**Table 2.** Barriers to AI use and AI access mode, overall, by country of residence, and by professional role. Barrier data: n=523 (1 non-respondent removed). Access mode data not mutually exclusive.

Variable and response option	All respondents (n=523), n (%)	Country of residence		*P* value^a^	
		United Kingdom (n=268), n (%)	United States (n=255), n (%)	Country of residence comparison	Professional role (physician/nurse/ other^b^) comparison^c^
Facilitating factors to use AI for work (select up to 2)					
Proof/evidence	227 (43.3)	112 (41.8)	115 (44.9)	—^d^	—
Organizational policy/support	156 (29.8)	91 (34.0)	65 (25.4)	—	—
Education/training	155 (29.6)	87 (32.5)	68 (26.6)	—	—
Stronger privacy protections	149 (28.4)	82 (30.6)	67 (26.2)	—	—
EHR^e^ integration	130 (24.8)	50 (18.7)	80 (31.3)	—	—
Time savings	116 (22.1)	60 (22.4)	56 (21.9)	—	—
Peer/leader endorsement	49 (9.4)	23 (8.6)	26 (10.2)	—	—
No interest in using AI	30 (5.7)	13 (4.9)	17 (6.6)	—	—
Already use AI enough	23 (4.4)	10 (3.7)	13 (5.1)	—	—
Other	10 (1.9)	6 (2.2)	4 (1.6)	—	—
Main factor limiting AI use in past 30 days (select 1)					
Concerned about accuracy/unreliable outputs	78 (14.9)	31 (11.6)	47 (18.4)	—	—
No access/not available in workplace	77 (14.7)	42 (15.7)	35 (13.7)	—	—
Not applicable; used AI with no major limitation	77 (14.7)	39 (14.6)	38 (14.9)	—	—
Prefer not to use AI (personal preference)	70 (13.4)	37 (13.8)	33 (12.9)	—	—
Concerned about privacy/security (PHI^f^)	65 (12.4)	33 (12.3)	32 (12.5)	—	—
Lack of training/not sure what to use	63 (12.0)	37 (13.8)	26 (10.2)	—	—
Not allowed by policy/compliance restrictions	45 (8.6)	26 (9.7)	19 (7.5)	—	—
Do not see a useful use case for my role	33 (6.3)	14 (5.2)	19 (7.5)	—	—
Concerned about medicolegal risk	8 (1.5)	2 (0.7)	6 (2.4)	—	—
Other	7 (1.3)	7 (2.6)	0 (0.0)	—	—
Where respondents access AI tools for work (select all that apply)					
Integrated into EHR/work systems	128 (24.4)	59 (22.0)	69 (27.0)	.19	.47^g^
Personal tool/account (not employer-provided)	210 (40.1)	106 (39.6)	104 (40.6)	.80	<.001^h^
Employer-approved standalone tool	134 (25.6)	66 (24.6)	68 (26.6)	.61	.96^g^
Not applicable/do not use AI tools for work	117 (22.3)	59 (22.0)	58 (22.7)	.86	.01^i^
Other access method/not listed	25 (4.8)	16 (6.0)	9 (3.5)	.18	.008

a *P* values by chi-square test comparing UK vs US respondents and physician/nurse/other groups.

b “Other” role includes pharmacists, psychologists, and social workers.

c Role-based *P* values shown for access items only (barrier distribution by role not formally analyzed as primary aim).

d Not applicable.

e EHR: electronic health record.

f PHI: personal health information.

g EHR-integrated and employer-approved access did not differ by role.

h “Personal tool access” by role: physician: 60.4% (67/111); nurse: 31.0% (87/281); other: 42.4% (56/132).

i “Not applicable/do not use AI tools for work” by role: physician: 11.7% (13/111); nurse: 26.7% (75/281); other: 22.0% (29/132).

## Discussion

In this cross-national survey, three-quarters of respondents reported recent workplace AI use. Use was concentrated in documentation, information retrieval, and clinical decision support rather than imaging or revenue-cycle tasks, suggesting earlier adoption where generative tools can be layered onto existing workflows with low implementation burden [4]. Overall adoption and breadth were similar across countries, although the tasks to which AI was applied differed [5]

Role-based differences were more pronounced. Physicians reported broader use and were substantially more likely to access AI through personal, non–employer-provided accounts. This pattern suggests both uneven workflow integration and a governance gap when clinician demand outpaces approved organizational access but may also reflect limited training in the risks of AI integration. Beyond role, unadjusted models identified other demographic predictors of breadth, suggesting that adoption disparities may intersect with broader structural and sociodemographic factors [6], although this merits further investigation in adjusted, adequately powered analyses.

The barriers do not support a single implementation solution. Reliability, access, privacy, and training concerns were all common, while medicolegal risk was rarely the main limitation [7]. This contrasts with literature emphasizing tool maturity, cost, regulatory uncertainty, reimbursement, and medicolegal governance [4]. In the United Kingdom, governance is heterogeneous. National Health Service organizations operate under General Data Protection Regulation constraints that restrict the transfer of patient data to external systems but rely largely on individual professional responsibility rather than formal monitoring [8], whereas some private settings impose stricter restrictions on AI use for clinical documentation. Thus, “no access” may reflect appropriate avoidance of unsanctioned tools, organizational prohibition, or unmet demand, each requiring a different implementation response [9].

Study limitations include online panel recruitment, self-reported 30-day behavior, heterogeneity within the “other HCP” group, and a cross-sectional design that cannot establish whether access drives use or motivated users seek it out. Overall, workplace AI use was common, but institutional access lagged behind adoption [10]. For HCPs without workplace access, near-term steps include using approved tools for lower-risk, non–public health information tasks where permitted, seeking AI training, and communicating unmet workflow needs to institutional leaders. In parallel, organizations should prioritize governed access, clear policies, privacy safeguards, and workflow integration to reduce reliance on personal tools [8,9].

## Supplementary material

10.2196/100681 Multimedia Appendix 1 Description of variables, demographic results, and univariate linear regression results for breadth of AI use.

## Abbreviations

EHR: electronic health record
HCP: health care professionals
STROBE: Strengthening the Reporting of Observational Studies in Epidemiology
