# Supplementary material for: Artificial Intelligence Use Among Health Care Professionals in the United States and United Kingdom: Cross-National Survey
Source: JMIR Form Res. 2026 Oct 19;10:e100681. doi: 10.2196/100681 (PMC13679857; doi:10.2196/100681)
Supplement: Supplementary file 1 [file formative-v10-e100681-s001.docx]

### **Multimedia Appendix 1: Supplementary Materials**

**Table S1. Variables and descriptions**

| Variable | Description | Type | Sample Values |
| --- | --- | --- | --- |
| Gender | The gender identity of the respondent. | Categorical | Woman (including Trans Female/Trans Woman), Man (including Trans Male/Trans Man) |
| Highest education level completed | The highest level of education attained by the respondent. | Ordinal/Categorical | Doctorate degree (PhD/other), Graduate degree (MA/MSc/MPhil/other), Undergraduate degree (BA/BSc/other), Technical/community college, High school diploma/A-levels |
| Years of work experience | The number of years the respondent has been working. | Ordinal/Categorical | 16+ years, 11-15 years, 6-10 years, 3-5 years, 1-2 years |
| Employment sector within medicine/healthcare | The respondent's employment sector within medicine/healthcare. | Categorical | Nurse, Doctor, Pharmacist, Psychologist, Social Worker |
| Age | The age of the respondent. | Numerical (Discrete) | Age range > 18 |
| Sex | The biological sex of the respondent. | Categorical | Female, Male |
| Ethnicity | A simplified categorization of the respondent's ethnicity. | Categorical | White, Asian, Black, Mixed, Other |
| Country of birth | The country where the respondent was born. | Categorical | United States, United Kingdom, Turkey, Nepal, Nigeria, Hong Kong, Portugal, Germany, Taiwan, Switzerland, Philippines |
| Country of residence | The country where the respondent currently resides. | Categorical | United States, United Kingdom |
| Nationality | The nationality of the respondent. | Categorical | United States, United Kingdom, Nepal, Nigeria, Germany, Hong Kong, Portugal, Taiwan |
| Language | The primary language of the respondent. | Categorical | English, Nepali, German, Cantonese, Portuguese, Tagalog-Filipino, Bengali |
| Employment status | The respondent's current employment status. | Categorical | Full-Time, Part-Time |
| What is your primary clinical specialty/area? | The respondent's main clinical specialty or area of practice. | Categorical | Primary care / general practice, Internal medicine subspecialty, Behavioral and mental health, Pharmacy, Emergency / urgent care, Care coordination and social services, Surgery (all surgical specialties), Pediatrics |
| In the past 30 days, which types of AI have you used for work? (Select all that apply) | Types of AI tools used for work in the past 30 days (multi-select). | Multi-select Categorical | Documentation (AI scribes/ambient note drafting; summaries; after-visit instructions), Clinical decision support (risk scores; alerts; recommendations), Literature search/ summarization, I have not used AI tools for work in the past 30 days, Data analysis / quality improvement, Imaging/ diagnostics support, Patient messaging drafts, Coding/ billing/ prior authorization help, Translation/ language support, Other AI use/ Not listed |
| In the past 30 days, what has been the main factor LIMITING your use of AI tools at work? (Select one) | The single main factor limiting AI use at work in the past 30 days. | Categorical | No access / not available in my workplace, Concerned about accuracy/unreliable outputs, Lack of training / Not sure what to use, Prefer not to use AI (personal preference), Concerned about privacy/security (PHI), Not allowed by policy / compliance restrictions, Do not see a useful use case for my role, Concerned about medico-legal risk, Not applicable; I used AI tools for work in the past 30 days and had no major limitations, Other reason/ not listed |
| What would make you MORE LIKELY to use AI at work? (Select up to 2) | Factors that would increase the likelihood of AI use at work (multi-select). | Multi-select Categorical | Proof of accuracy/safety for my tasks, Time savings demonstrated, Clear organizational policy, Education and training, Strong privacy safeguards, Electronic Medical Records (EMR) integration, Peer/leadership endorsement, Nothing; I have no interest, Nothing; I already use AI as much as I want, Other factor/ not listed |
| Where do you access AI tools for work? (Select all that apply) | The location or method of accessing AI tools for work (multi-select). | Multi-select Categorical | Integrated into EHR/work systems, Personal tool/account (not employer-provided), Employer-approved standalone tool, Not applicable; I do not use AI tools for work, Other access method / not listed |

**Table S2. Demographic results**

| **A. RESPONDENT CHARACTERISTICS** | **Total**  **N (%)** | **United Kingdom**  **N (%)** | **United States**  **N (%)** |
| --- | --- | --- | --- |
| **Country of residence** |  |  |  |
| United Kingdom | 268 (51.1) | 268 (100) | NA |
| United States | 256 (48.9) | NA | 256 (100) |
| **Gender** |  |  |  |
| Man (including Trans Male/Trans Man) | 137 (26.1) | 64 (23.9) | 73 (28.5) |
| Non-binary | 7 (1.3) | 1 (0.4) | 6 (2.4) |
| Woman (including Trans Female/Trans Woman) | 380 (72.5) | 203 (75.7) | 177 (69.1) |
| **Sex** |  |  |  |
| Female | 386 (73.7) | 204 (76.1) | 182 (71.1) |
| Male | 138 (26.3) | 64 (23.9) | 74 (28.9) |
| **Age** |  |  |  |
| 18-29 | 107 (20.4) | 48 (17.9) | 59 (23.0) |
| 30-44 | 275 (52.5) | 151 (56.3) | 124 (48.4) |
| 45-59 | 108 (20.6) | 51 (19.1) | 57 (22.3) |
| 60 or older | 34 (6.5) | 18 (6.7) | 16 (6.3) |
| **Highest education level completed** |  |  |  |
| Secondary education (eg, GED/GCSE) | 3 (0.6) | 3 (1.1) | 0 (0.0) |
| High school diploma/A-levels | 26 (5.0) | 15 (5.6) | 11 (4.3) |
| Technical/community college | 39 (7.4) | 7 (2.6) | 32 (12.5) |
| Undergraduate degree (BA/BSc/other) | 228 (43.5) | 122 (45.5) | 106 (41.4) |
| Graduate degree (MA/MSc/MPhil/other) | 157 (30.0) | 105 (39.2) | 52 (20.3) |
| Doctorate degree (PhD/other) | 71 (13.5) | 16 (6.0) | 55 (21.5) |
| **Years of work experience** |  |  |  |
| 1-2 years | 24 (4.6) | 9 (3.4) | 15 (5.8) |
| 3-5 years | 72 (13.7) | 32 (11.9) | 40 (15.6) |
| 6-10 years | 130 (24.8) | 58 (21.6) | 72 (28.1) |
| 11-15 years | 89 (17.0) | 57 (21.3) | 32 (12.5) |
| 16+ years | 209 (39.9) | 112 (41.8) | 97 (38.0) |
| **Employment sector within medicine/healthcare** |  |  |  |
| Doctor | 111 (21.2) | 62 (23.1) | 49 (19.1) |
| Nurse | 281 (53.6) | 139 (51.9) | 142 (55.5) |
| Pharmacist | 73 (13.9) | 34 (12.7) | 39 (15.2) |
| Psychologist | 29 (5.5) | 17 (6.3) | 12 (4.7) |
| Social Worker | 30 (5.7) | 16 (6.0) | 14 (5.5) |
| **Ethnicity** |  |  |  |
| Asian | 65 (12.4) | 41 (15.3) | 24 (9.4) |
| Black | 46 (8.8) | 25 (9.3) | 21 (8.2) |
| Mixed | 24 (4.6) | 7 (2.6) | 17 (6.6) |
| Other | 10 (1.9) | 5 (1.9) | 5 (2.0) |
| Prefer not to say | 3 (0.6) | 1 (0.4) | 2 (0.8) |
| White | 376 (71.8) | 189 (70.5) | 187 (73.0) |
| **Employment status** |  |  |  |
| Full-Time | 390 (74.4) | 194 (72.4) | 196 (76.6) |
| Part-Time | 134 (25.6) | 74 (27.6) | 60 (23.4) |
| **Primary clinical specialty/area (N=523)** |  |  |  |
| Behavioral and mental health | 78 (14.9) | 46 (17.2) | 32 (12.6) |
| Care coordination and social services | 35 (6.7) | 20 (7.5) | 15 (5.9) |
| Emergency/urgent care | 49 (9.4) | 26 (9.7) | 23 (9.0) |
| Internal medicine subspecialty | 54 (10.3) | 32 (11.9) | 22 (8.6) |
| OB/GYN | 20 (3.8) | 5 (1.9) | 15 (5.9) |
| Pediatrics | 33 (6.3) | 14 (5.2) | 19 (7.4) |
| Pharmacy | 60 (11.5) | 26 (9.7) | 34 (13.3) |
| Primary care/general practice | 153 (29.3) | 83 (31.0) | 70 (27.5) |
| Radiology/imaging | 4 (0.8) | 1 (0.3) | 3 (1.2) |
| Surgery (all surgical specialties) | 37 (7.1) | 15 (5.6) | 22 (8.6) |

**Table S3**. **Univariate Linear Regression: Predictors of AI Usage Breadth Score**

|  | Univariate B-coefficient (95% CI) | p-value |
| --- | --- | --- |
| Gender |  |  |
| Male | Reference |  |
| Non-binary | 0.58 (-0.47, 1.62) | 0.28 |
| Female | -0.28 (-0.55, -0.01) | 0.04 |
| Education |  |  |
| High school diploma | Reference |  |
| Secondary education | 0.08 (-1.52, 1.69) | 0.93 |
| Technical/community college | 0.38 (-0.29, 1.06) | 0.26 |
| Undergraduate degree | 0.37 (-0.17, 0.93) | 0.18 |
| Graduate degree | 0.85 (0.29, 1.40) | <0.01 |
| Doctorate degree | 1.03 (0.43, 1.64) | <0.01 |
| Years of work experience |  |  |
| 1-2 years | Reference |  |
| 3-5 years | 0.07 (-0.57, 0.70) | 0.83 |
| 6-10 years | 0.45 (-0.15, 1.05) | 0.14 |
| 11-15 years | 0.38 (-0.24, 1.00) | 0.23 |
| 16+ years | 0.50 (-0.07, 1.08) | 0.09 |
| Age | 0.01 (-0.01, 0.02) | 0.15 |
| Male sex | 0.28 (0.01, 0.55) | 0.04 |
| Role |  |  |
| Doctor | Reference |  |
| Nurse | -0.63 (-0.92, -0.33) | <0.01 |
| Other | -0.61 (-0.94, -0.26) | <0.01 |
| Race |  |  |
| Asian | Reference |  |
| Black | 0.69 (0.17, 1.21) | 0.01 |
| Mixed | 0.08 (-0.56, 0.72) | 0.81 |
| Other/not answer | 0.08 (-0.74, 0.89) | 0.85 |
| White | -0.02 (-0.38, 0.34) | 0.90 |
| Country of Residence |  |  |
| United Kingdom | Reference |  |
| United States | 0.19 (-0.04, 0.42) | 0.11 |
| English as primary language | -0.05 (-0.43, 0.33) | 0.81 |
| Full time employment | 0.37 (0.10, 0.64) | 0.01 |
| Specialty |  |  |
| Primary care/general practice | Reference |  |
| Behavioral Health | -0.31 (-0.68, 0.06) | 0.10 |
| Care Coordination/Social Services | 0.43 (-0.12, 0.97) | 0.12 |
| Emergency Medicine | 0.49 (0.01, 0.98) | 0.05 |
| Internal Medicine | 0.28 (-0.19, 0.76) | 0.24 |
| Obstetrics/gynecology | 0.10 (-0.57, 0.78) | 0.76 |
| Pediatrics | -0.41 (-0.97, 0.15) | 0.15 |
| Pharmacy | 0.01 (-0.46, 0.47) | 0.98 |
| Radiology | -0.35 (-1.72, 1.03) | 0.62 |
| Surgery | 0.01 (-0.53, 0.54) | 0.99 |

AI usage score = sum of AI tool types used in the past 30 days (range 0-9, excluding "not used" response option). Each row represents a separate univariate regression model; N ranged from 523 to 524 because of missing data for some covariates. CI, confidence interval.
